# CoGA: An R Package to Identify Differentially Co-Expressed Gene Sets by Analyzing the Graph Spectra

**DOI:** 10.1371/journal.pone.0135831

**Published:** 2015-08-27

**Authors:** Suzana de Siqueira Santos, Thais Fernanda de Almeida Galatro, Rodrigo Akira Watanabe, Sueli Mieko Oba-Shinjo, Suely Kazue Nagahashi Marie, André Fujita

**Affiliations:** 1 Department of Computer Science, Institute of Mathematics and Statistics, University of São Paulo, São Paulo, Brazil; 2 Department of Neurology, School of Medicine, University of São Paulo, São Paulo, Brazil; Leibniz-Institute for Farm Animal Biology (FBN), GERMANY

## Abstract

Gene set analysis aims to identify predefined sets of functionally related genes that are differentially expressed between two conditions. Although gene set analysis has been very successful, by incorporating biological knowledge about the gene sets and enhancing statistical power over gene-by-gene analyses, it does not take into account the correlation (association) structure among the genes. In this work, we present CoGA (Co-expression Graph Analyzer), an R package for the identification of groups of differentially *associated* genes between two phenotypes. The analysis is based on concepts of Information Theory applied to the spectral distributions of the gene co-expression graphs, such as the spectral entropy to measure the randomness of a graph structure and the Jensen-Shannon divergence to discriminate classes of graphs. The package also includes common measures to compare gene co-expression networks in terms of their structural properties, such as centrality, degree distribution, shortest path length, and clustering coefficient. Besides the structural analyses, CoGA also includes graphical interfaces for visual inspection of the networks, ranking of genes according to their “importance” in the network, and the standard differential expression analysis. We show by both simulation experiments and analyses of real data that the statistical tests performed by CoGA indeed control the rate of false positives and is able to identify differentially co-expressed genes that other methods failed.

## Introduction

Many biomedical studies aim to understand the differential regulation of genes (e.g. between diseased and healthy people) by analyzing gene expression data. An often used approach is the test of equality in the average expression of single genes between two populations with different phenotypes.

Alternatively, gene set analysis methods such as the well-known GSEA [1], test the differential expression of *sets* of related genes (pathways). The main advantage is that they enhance statistical power and aggregate prior biological knowledge. A motivation to test pathways is based on the idea that complex diseases are rarely consequence of an abnormality in a single gene, but a result of changes in a set of related ones. Despite their success, the GSEA and similar approaches do not identify important classes of differentially regulated pathways, such as groups of differentially co-expressed genes.

The co-expression of two genes is the correlation between their expression levels. If the correlation structure (also known as co-expression graph) among the genes of a group in one phenotype is different from that in another, this group is called differentially co-expressed. Differential co-expression of genes may provide information about changes in the gene regulatory networks of different phenotypes.

It is important to clarify that differentially co-expressed genes are not necessarily differentially expressed [2–6]. For instance, mutations on the activation domain of transcription factors (TFs) can change the TF behavior without altering their expression levels [7]. Furthermore, the regulatory activities of a gene product can be affected by post-translational modifications without changing the gene expression levels [6]. Thus, the differential co-expression analysis complements the differential expression analysis.

Several approaches were developed to measure and test the differential co-expression of genes. Examples include methods that address the problem for each gene pair [8, 9], methods that find gene modules that are differentially co-expressed [7, 10–13], and methods that test the differential co-expression for a predefined collection of gene sets [14, 15]. Those methods vary in how they quantify co-expression between genes, how they measure changes in the co-expression of a group of genes, and how they cluster the genes. In this work, we address the problem of measuring the differential co-expression of a given gene set, which is also addressed by the GSCA [14] and GSNCA methods [15].

One of the main challenges of measuring differential co-expression of a given gene set is the fact that searching for an exact common structure between two co-expression graphs is not effective to compare the behavior of biological pathways, as their structure may vary across time and across systems from the same biological class. The GSCA method compares the co-expression of a gene set between two phenotypes by summing the changes in the correlation of each gene pair of the set. The main limitation of that approach is that it does not take into account the structure of the gene co-expression graph. To address that limitation, Rahmatallah *et al* proposed the GSNCA method [15], which is based on the idea that biological systems tend to be more affected by changes in the activities of “important” genes than isolate alterations in the gene interactions [16]. The GSNCA considers that the “importance” of a gene is proportional to the sum of the “importance” of the other genes weighted by the cross-correlations. To find dysfunctional pathways, the GSNCA tests the changes in the gene “importance” (centrality) between two biological conditions, identifying classes of differentially co-expressed gene sets that were not detected by the GSCA.

The GSNCA measure of centrality (also known as eigenvector centrality) is one among several measures of graph structural features, such as the clustering coefficient, the shortest path length, and the betweenness, closeness and degree centralities. Some of those features are explored by tools such as the WGCNA [13] and the Cytoscape [17]. However, those tools do not carry out statistical tests for the identification of changes in the structural features. Furthermore, many structural properties of gene co-expression graphs remain underexplored.

To identify dysfunctional pathways, we want to compare structural features that are shared by networks belonging to the same biological class but that are distinct between different classes. The spectrum of a graph, defined as the set of eigenvalues of its adjacency matrix, describes several features of the graph, such as its diameter, number of walks, and cliques. Takahashi *et al* [18] proposed that the graph spectrum distribution is a better characterization of a graph class when compared to other commonly used measures (e.g. number of edges, average path length and clustering coefficient). Based on the spectral characterization of a graph, Takahashi *et al* [18] introduced concepts of Information Theory for graphs, such as the spectral entropy and the Jensen-Shannon divergence between spectral densities. The former measures the amount of uncertainty associated with a graph, while the latter is used to discriminate classes of graphs. The measures proposed by Takahashi *et al* have successfully identified structural changes in brain networks [18]. In this work, we present a tool that adapts the spectral entropy and Jensen-Shannon divergence statistical tests for gene co-expression graphs.

Our proposed tool is CoGA (Co-expression Graph Analyzer), an R package that constructs co-expression graphs and identifies differentially co-expressed gene sets by statistically testing the equality in the spectral distribution [18] of the co-expression (sub-)graphs. It also includes tests for other features of the graphs, such as the graph spectral entropy [18], a variety of centralities, clustering coefficients, and degree distribution. CoGA differs from other differential co-expression analysis tools in two ways: (i) it statistically tests the significance of network alterations for a large variety of structural features; and (ii) it includes further analysis, such as network visualization, gene scores, and single gene differential expression analysis. By performing Monte Carlo experiments and applying the proposed method in biological data from glioma tissues, we show that the CoGA test effectively controls the rate of false positives and also identifies dysfunctional pathways that other tools did not detect. In other words, CoGA complements both GSCA and GSNCA.

## Materials and Methods

The CoGA R package compares gene co-expression networks in terms of their structural properties. In the following subsections we explain the construction of co-expression networks (graphs), the graph spectral analysis, and the package main features.

### Construction of gene co-expression networks

An undirected graph is an ordered pair *G* = (*V*, *E*) that contains a set of vertices (*V*), and a set of edges (*E*), which connect the elements of *V*. Each edge *e* ∈ *E* is an unordered pair *e* = {*v*_*i*_, *v*_*j*_}, such that *v*_*i*_ and *v*_*j*_ are two distinct nodes that belong to *V*.

A gene co-expression network is an undirected graph, where each vertex corresponds to a gene and an edge connecting a pair of vertices indicates a relationship between the expression levels of the corresponding genes. In this context, the association represented by an edge corresponds to the statistical dependence among the gene expression levels. To measure and detect monotonic dependence, the CoGA package includes the correlations and dependence tests of Pearson [19], Spearman [20], and Kendall [21].

Given a measure of statistical dependence (Pearson’s, Spearman’s or Kendall’s correlation coefficients), CoGA provides three scales for measuring the association degree between the activities of two genes: the absolute correlation coefficient, one minus the p-value of the dependence test, and one minus the adjusted p-value by the False Discovery Rate method [22] for multiple testing. Each association degree is a real number varying from 0 to 1.

The user can choose between *unweighted* and *weighted* network. The former is a graph with edges selected according to an association degree threshold defined by the user. Alternatively, the software generates a full graph with edges weighted by the association degrees between the gene expression levels.

In both simulations and application to actual biological data, we consider co-expression graphs in which the edges are weighted by one minus the Spearman’s p-value adjusted for multiple testing. We describe each graph by its spectral distribution, as detailed in the next section.

### Graph spectral analysis

Let *G* = (*V*, *E*) be an undirected graph with *n*_*V*_ vertices. We represent the weight of the edge that connects the vertices *v*_*i*_, *v*_*j*_ ∈ *V* by *w*_*ij*_. Consider that if *G* is unweighted, then *w*_*ij*_ = 1 for all edge *e* = {*v*_*i*_, *v*_*j*_} that belongs to *E*. We define the *adjacency matrix* of *G* to be a *n*_*V*_ × *n*_*V*_ matrix *A*, such that:

$$\begin{array}{cc} A_{ij} & =w_{ij}\text{,}\;\text{if}\;v_{i}\;\text{and}\;v_{j}\;\text{are}\;\text{connected}\;\text{by}\;\text{an}\;\text{edge}.\\ A_{ij} & =0\text{,}\;\text{if}\;\text{there}\;\text{is}\;\text{no}\;\text{edge}\;\text{connecting}\;v_{i}\;\text{and}\;v_{j}. \end{array}$$

The *spectrum* of *G* is the set of eigenvalues of its adjacency matrix *A* [18]. It describes many structural properties of a graph, such as the number of walks, diameter, and cliques [23]. Based on the graph spectrum distribution, Takahashi *et al*. [18] introduced the concepts of spectral entropy to measure the amount of uncertainty associated with a graph, and the Jensen-Shannon divergence between spectral densities as a distance between graphs. We explain such concepts in the next sections.

#### Spectral density

A graph model is an algorithm that generates graphs according to a probability law. Given a graph model, let *g* denote the family of all graphs generated by the model, each one containing *n*_*V*_ vertices. The *spectral density* of *g* is the probability density function of the spectra of the graphs that belong to *g*.

Let *δ* and “〈〉” denote the Dirac’s delta, and the expectation according to the probability law of *g*, respectively. Formally, the *spectral density* of the family of graphs *g* is defined as [18]:

$$\rho \left(\lambda \right)=\lim_{n_{V}\to \infty }\left\langle \frac{1}{n_{V}}\sum_{j=1}^{n}\delta (\left(\lambda -\lambda_{j}\right)/\sqrt{n_{V}})\right\rangle .$$

In real systems, the spectral density is unknown. To estimate the probability density function from the observed spectrum of a given graph, CoGA uses the Gaussian Kernel estimate implemented by the function density from the R base package. The user can choose between the Sturges’ [24] and the Silverman’s criteria [25] to define the Kernel bandwidth for the Gaussian Kernel estimate. For both simulation and actual data analysis, we used the Sturges’ criterion.

#### Spectral entropy

In information theory, the entropy of a random variable *X* measures the amount of uncertainty associated with the value of *X*. For a graph, the entropy quantifies the randomness of its structure.

Formally, the spectral entropy is defined as follows. Let *g* be a family of graphs generated according to a probability law, and *ρ* denote the spectral density of *g*. The spectral entropy [18] of *G* is:

$$H\left(\rho \right)=-\int_{-\infty }^{+\infty }\;\rho (\lambda )\log\rho (\lambda )d\lambda ,$$

where 0 log 0 = 0.

#### Kullback-Leibler divergence

While the entropy quantifies the uncertainty associated with a random variable, the Kullback-Leibler (KL) divergence measures the information lost when a probability distribution is used to approximate another. For graphs, we can use the KL divergence to discriminate spectral distributions and also to select the graph model that best describes the observed graph. Formally, we define the *KL* divergence between graphs as follows.

Let *g*_1_ and *g*_2_ be two graph families with spectral densities *ρ*_1_ and *ρ*_2_, respectively. If the support of *ρ*_2_ contains the support of *ρ*_1_, then the KL divergence between *ρ*_1_ and *ρ*_2_ is [18]:

$$KL\left(\rho_{1}|\rho_{2}\right)=\int_{-\infty }^{+\infty }\;\rho_{1}\left(\lambda \right)\log\frac{\rho_{1}\left(\lambda \right)}{\rho_{2}\left(\lambda \right)}d\lambda ,$$

where 0 log 0 = 0 and *ρ*_2_ is called the reference measure.

If the support of *ρ*_2_ does not contain the support of *ρ*_1_, then *KL*(*ρ*_1_∣*ρ*_2_) = +∞.

The KL divergence is non-negative, and it is zero if and only if *ρ*_1_ and *ρ*_2_ are equal. For many cases, *KL*(*ρ*_1_∣*ρ*_2_) and *KL*(*ρ*_2_∣*ρ*_1_) are different when *ρ*_1_ and *ρ*_2_ are not equal, i.e. *KL* is an asymmetric measure.

#### Jensen-Shannon divergence

The Jensen-Shannon (JS) divergence is a symmetric alternative to the KL divergence. Let $\rho_{M}=\frac{1}{2}(\rho_{1}+\rho_{2})$, then the JS divergence between two spectral densities *ρ*_1_ and *ρ*_2_ is defined as [18]:

$$JS\left(\rho_{1},\rho_{2}\right)=\frac{1}{2}KL\left(\rho_{1}|\rho_{M}\right)+\frac{1}{2}KL\left(\rho_{2}|\rho_{M}\right).$$

We can interpret the JS divergence as a distance between two graphs. The square root of the measure has all mathematical properties of a metric: (i) is zero if and only if *ρ*_1_ and *ρ*_2_ are equal, (ii) is symmetric, (iii) is non-negative, and (iv) satisfies the triangle inequality.

### Description of the CoGA package

**Fig 1:**
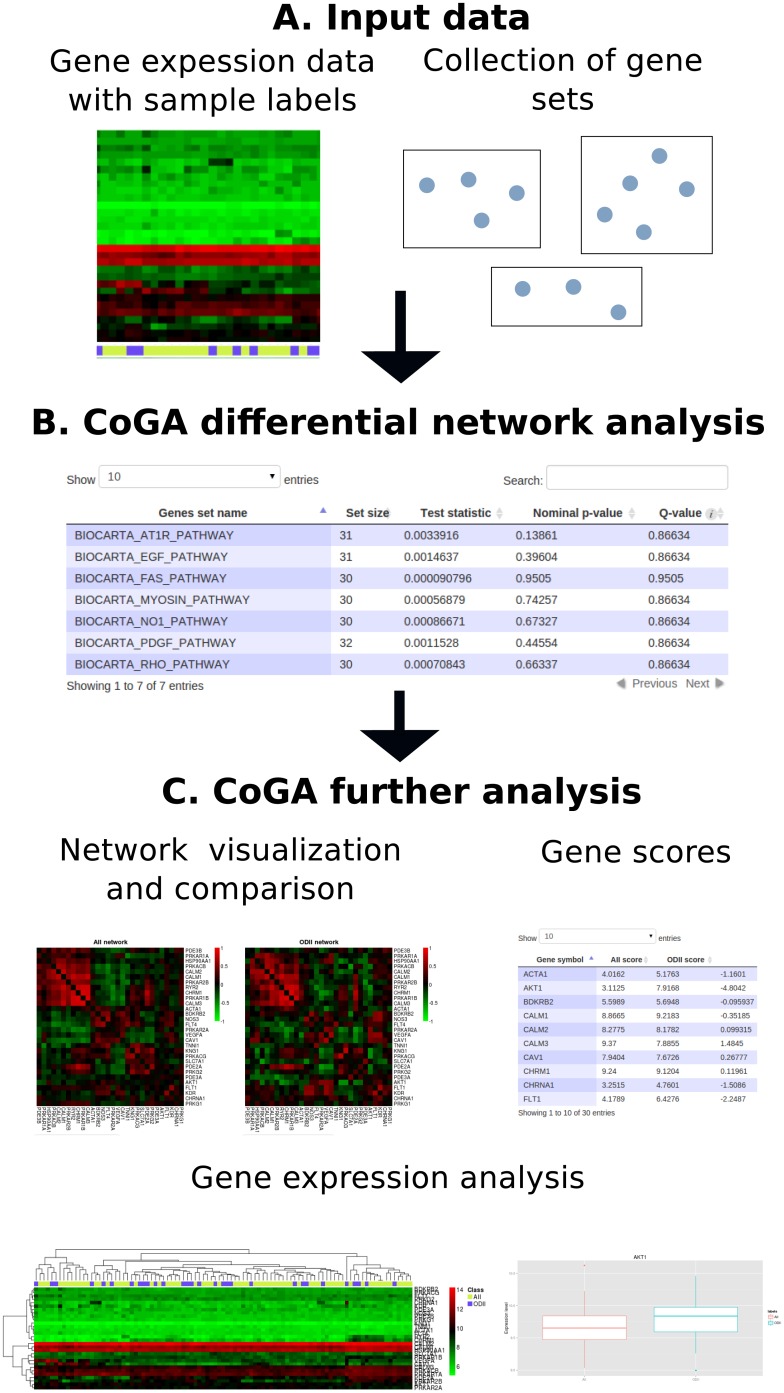
CoGA Overview: (A) input data, (B) CoGA differential network analysis, and (C) CoGA further analysis. The CoGA package receives as input data a gene expression matrix, the sample labels, and a collection of gene sets. Then, it constructs a gene co-expression sub-graph for each gene set, and tests the equality in the network structural features between two biological conditions (B). The software allows the user to further analyze each gene set (C) by visualizing the gene co-expression graphs, ranking the genes according to their “importance” in the gene set network, and performing standard single gene differential expression analysis.

The CoGA package is a tool with a graphical interface to analyze gene co-expression networks. It receives gene expression data and a predefined collection of gene sets from which it performs the differential network analysis. The software also includes further analyses of a gene set, such as network visualization, the centralities of the genes that belong to the set and the standard single gene differential expression analysis, as shown in Fig 1. In the next paragraphs we describe briefly the input, output and main features of the package. For a detailed tutorial and manual we refer the user to the page www.ime.usp.br/˗suzana/coga.

#### Input

CoGA receives three files as input: one with the pre-processed gene expression data, one with labels indicating the association between the sample and its phenotype, and another one containing the pre-defined collection of gene sets (e.g. sets of genes belonging to same pathways or sharing the same Gene Ontology terms). If the dataset has not been collapsed to gene symbols yet, it is necessary to upload a fourth file with the annotation data (i.e. a table that indicates the correspondence between probe sets and genes).

For examples of gene set collections and microarray annotation data, we suggest to use files from the Molecular Signature Database (MSigDB) (http://www.broadinstitute.org/gsea/msigdb/index.jsp) and the Broad ftp site (ftp://gseaftp.broadinstitute.org/pub/gsea/annotations), respectively. Both databases are freely available for download.

#### Differential network analysis

The differential network analysis is divided in two parts. First, given a gene set, CoGA constructs one network for each phenotype by calculating the pairwise correlations between the expressions of the genes in the set. Then, it computes a statistic to compare the structural properties of the inferred graphs between the two phenotypes. To obtain a p-value for the statistic, both steps are repeated several times using random permutations of the sample labels. Finally, CoGA computes the p-value for each gene set.

Let Θ be a measure of the difference (“distance”) between the structural properties of two graphs. CoGA tests *H*_0_ : Θ = 0 against *H*_1_ : Θ > 0. For estimating Θ, CoGA implements an adaptation of the Jensen-Shannon divergence between the graph spectrum distributions proposed by Takahashi *et al*, [18]. Other measures of the differences between graph structural features implemented by CoGA are the Jensen-Shannon divergence between the degree distributions, the Euclidian distance (adjusted for the gene set size) between the node centralities and between the clustering coefficients, and the absolute difference between the average shortest path lengths and between the spectral entropies.

#### Output

CoGA returns a table containing the name and size of each gene set, the statistics used in the test, permutation-based p-values, and adjusted p-values by False Discovery Rate method [22] for multiple tests.

#### Other features

CoGA features include an interface to visually inspect alterations in the co-expression networks, a list of the differences in the pairwise correlations, a table of gene set properties (e.g. spectral entropy, average node centrality, average clustering coefficient, and average shortest path length) in each phenotype, a ranking of the gene centralities and local clustering coefficients, and single gene differential expression analysis.

### Implementation

CoGA was implemented in R (http://cran.r-project.org/) and requires the following packages to run: (i) shiny, shinyBS, yaml, whisker and RJSONIO for browser user interface; (ii) igraph to compute graph topological properties; (iii) WGCNA to collapse probe sets to gene symbols; (iv) ggplot2, pheatmap, and RColorBrewer for plotting; and (v) Hmisc and psych for graph inference. For some graphical interface features, we used code from: rCharts (https://github.com/ramnathv/rCharts) and shinyIncubator (https://github.com/rstudio/shiny-incubator).

### Example dataset

To illustrate a CoGA input dataset, we downloaded an Affymetrix Human Genome U133 plus 2.0 microarray dataset from two subtypes of brain cancer: 65 astrocytomas grade II (AII) and 30 oligodentrogliomas grade II (ODII) microarrays available at the REMBRANDT database (https://caintegrator.nci.nih.gov/rembrandt). Our choice was motivated by the fact that the differential diagnosis between AII and ODII is a difficult task due to their very similar phenotypes.

We pre-processed the raw data (CEL files) with the RMA (*Robust Multichip Average*) [26] method for adjustment of background, normalization, and summarization. To arrange probes into probe sets based on updated genome and transcriptome information, we used the Brainarray [27] custom CDF file (version 18.0.0, ENTREZG), which collapsed our dataset to 19,674 gene symbols.

## Results and Discussion

In this section we show both simulation experiments and analyses of biological data to evaluate the performance of CoGA.

### Control of the false positive rate

To validate the effective control of the rate of false positives, we applied the spectral distribution test in 1,000 artificially generated biological datasets. To generate those datasets, we put together the data from 65 astrocytoma grade II and 30 oligodentroglioma grade II microarrays into one dataset. Then, for simulating the null hypothesis (the networks come from the same population), a resample of 65 microarrays and another one of 30 microarrays were taken at random, with replacement, from this pooled dataset to make new realizations of each phenotype group. Finally, we applied the proposed test to different gene set sizes, ranging from 20 to 100. Small sets (e.g. smaller than 20 genes) may interfere the estimation of the network features, while large sets (e.g. larger than 1,000 genes) may lead to a very high computational cost (depending on the specification of the user’s machine). Furthermore, the sample size must be large enough (our empirical studies suggest at least 20 samples, but this number also depends on the data variance, noise, and how many tests will be performed) to infer the co-expression among genes.

Each permutation test used 1,000 random resamplings for this experiment. Under the null hypothesis, we expect that the proportion of false positives will be less than or equal to the significance level of the test (p-value threshold for rejecting *H*_0_). In our simulation experiments, for a p-value threshold (*α*) of 1, 5, and 10% (Table 1), about 1, 5, and 10% of the tests, respectively, incorrectly rejected the null hypothesis. Therefore, the rate of false positives is indeed controlled as expected.

**Table 1:** Observed false positive rate. Proportion of incorrectly rejected null hypotheses for different significance levels (*α* = 0.01, 0.05, 0.1) and different gene set sizes (*n*_*V*_ = 20, 40, 100).

*α*	*n*_*V*_		
	20	40	100
0.01	0.008	0.010	0.010
0.05	0.042	0.041	0.057
0.10	0.089	0.101	0.118

### Comparisons with other methods

To compare with CoGA, we selected both GSCA [14] and GSNCA [15] because they address the same problem: to identify differentially co-expressed gene sets between two biological conditions. To evaluate the performance of the three methods, we adapted GSCA and GSNCA to be in accordance with CoGA co-expression graph construction (i.e. to measure the association between the expression levels, we replaced the Pearson’s correlation used in both GSCA and GSNCA by one minus Spearman’s p-value adjusted for multiple testing). Then we carried out Monte Carlo simulations, and analyzed a real dataset.

#### Simulation experiments

To evaluate the statistical power of CoGA, GSCA, and GSNCA methods, we generated data as follows. First we took at random 80 microarrays from the pooled dataset containing data from astrocytoma grade II (AII) and oligodendroglioma grade II (ODII) microarrays. Then we split that resample of 80 microarrays into two parts of size 40, each one representing a phenotype group that will be compared. For each phenotype group, we measured the co-expression among 50 genes that were taken at random. To change the co-expression of some of the 50 selected genes, we permuted the expression levels of a proportion *γ* of genes in only one group of microarrays. Thus, the resulting co-expression graphs generated by this process will be different. We repeated this procedure 1,000 times for different proportions of altered genes (*γ*), varying from 0.05 to 0.5 in steps of 0.05.

To summarize the empirical power of the tests (proportion of rejected null hypotheses) for different significance levels (*α*), we measured the areas under the ROC curves. The ROC curve is drawn over a two-dimensional plot, where the *x*-axis corresponds to the significance levels of the tests and the *y*-axis corresponds to the proportion of rejected null hypotheses (empirical statistical power). Then the area under the ROC curve (AUC) is a summary of the empirical power of the tests for different significance levels. Under the alternative hypothesis, we want the AUC to be larger than 0.5 and as close as possible to 1. In Table 2, we show the AUC for *γ* = 0.05, 0.1, 0.15, 0.2, 0.25, 0.3, 0.5. As expected, for all methods, the statistical power increases with the increasing of *γ* (proportion of altered genes). Thus, all methods were able to discriminate different graphs.

**Table 2:** Evaluation of the statistical power of the tests. Areas below the ROC curves for different proportions of altered genes (*γ*), varying from 0.05 to 0.5. The ROC curves were constructed for the CoGA, GSCA, and GSNCA methods.

Method	*γ*						
	0.05	0.1	0.15	0.2	0.25	0.3	0.5
CoGA	0.505	0.663	0.746	0.869	0.934	0.968	0.999
GSCA	0.587	0.786	0.848	0.941	0.981	0.995	0.999
GSNCA	0.588	0.784	0.831	0.910	0.956	0.974	0.994

It is important to highlight that the performance of the methods in a specific dataset will depend on the structural changes occurring in the co-expression networks. We show in Table 3 the number of resamples for which the differential co-expression was detected only by one of the methods considering different significance levels (*α* = 0.01, 0.05, 0.1). Each method uniquely identified resamples with differentially co-expressed genes for all scenarios, except when *γ* = 0.5 (because almost all null hypotheses were rejected in that scenario). Thus our results suggest that the GSCA, GSNCA, and CoGA methods complement each other.

**Table 3:** Comparison of the method findings in simulation experiments. Number of generated datasets for which only one of the three methods (CoGA, GSCA, and GSNCA) detected differential co-expression. The proportion *γ* of genes whose expression levels were permuted in only one of the two conditions being tested varies from 0.05 to 0.5. We generated 1,000 datasets and considered different significance levels (*α* = 0.01, 0.05, 0.1) for rejecting the null hypothesis.

Method	*α*	*γ*						
		0.05	0.1	0.15	0.2	0.25	0.3	0.5
CoGA	0.01	6	32	36	54	20	8	0
	0.05	32	74	83	40	19	5	0
	0.1	59	87	80	36	15	3	0
GSCA	0.01	14	63	80	146	145	130	2
	0.05	47	96	107	84	48	22	0
	0.1	63	93	87	48	27	8	0
GSNCA	0.01	13	37	53	76	50	20	0
	0.05	50	113	106	58	25	7	0
	0.1	77	124	94	50	17	4	0

#### Biological data analysis

To compare the CoGA, GSCA, and GSNCA performances in a real dataset, we analyzed the original dataset containing 65 microarrays from astrocytoma grade II (AII) and 30 microarrays from oligodendroglioma grade II (ODII) using each method. Those methods require a collection of gene sets, which corresponds to the sub-networks for the differential co-expression analysis. In this comparative analysis, the collection corresponds to the canonical pathways from the MSigDB v4.0. After setting the minimum gene set size to 20, only 850 of 1,320 gene sets remained for the analyses.

**Fig 2:**
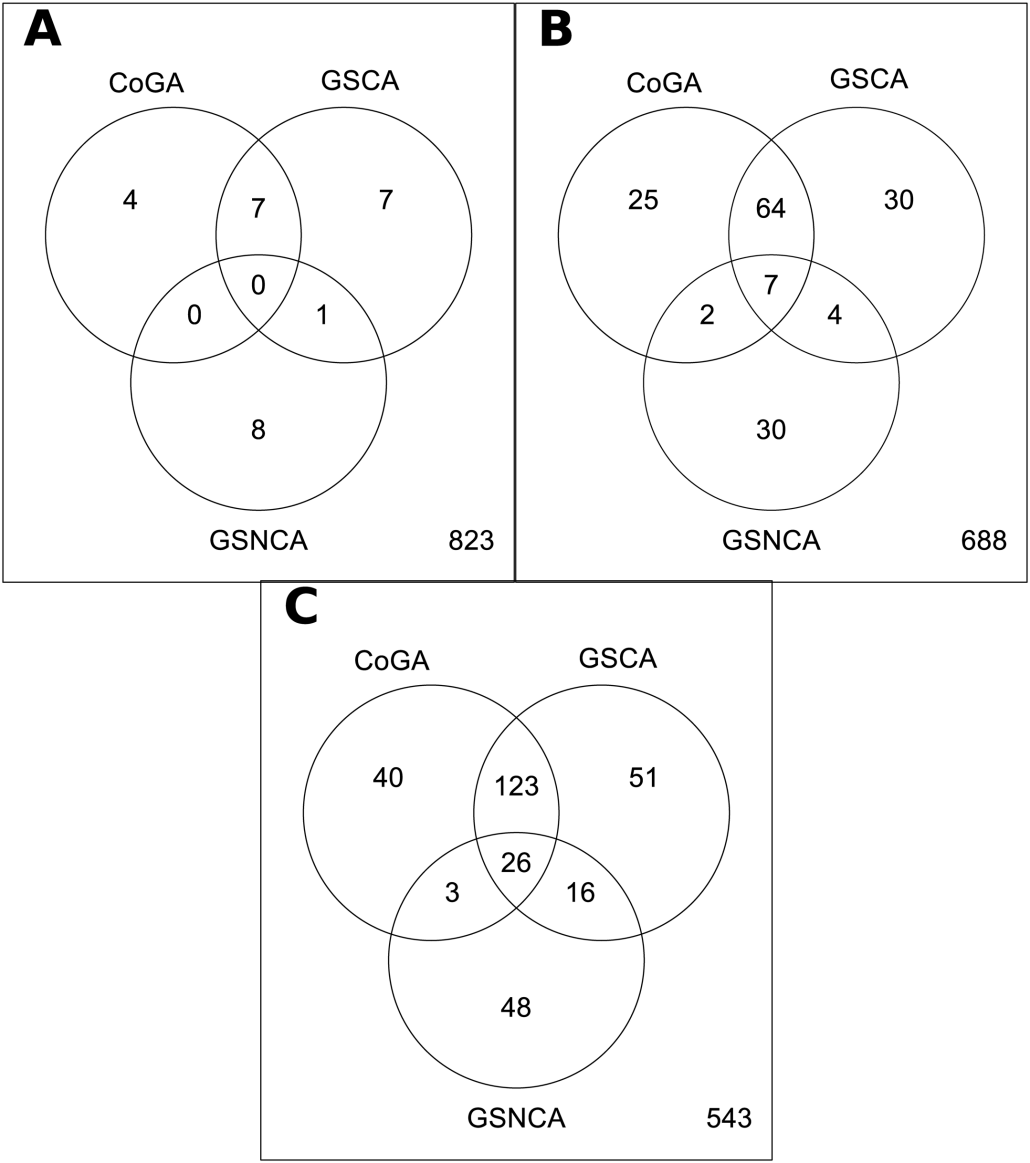
Venn diagrams of the gene sets co-identified by the methods. Each diagram shows the number of gene sets co-identified by the Spectral distribution test from the CoGA package, and the GSCA and GSNCA methods. In (A), (B), and (C) the significance level of the tests is set to 0.01, 0.05, and 0.1, respectively.

For each permutation test, we set the number of random resamples to 10,000. We show the resulting p-values for all gene sets in S1 Table. In Fig 2, we show Venn diagrams of the gene sets co-identified by the methods for different significance levels (*α* = 0.01, 0.05, 0.10). When the significance level (*α*) is 0.01, the CoGA package identified four sets that were not detected by the other methods. For *α* = 0.05 and *α* = 0.1, the number of sets identified only by CoGA is 25 and 40, respectively. Then, the CoGA method can identify sets that were not detected by the GSCA and the GSNCA tests.

In Table 4, we show the gene sets that were identified only by one of the three methods for a significance level (*α*) of 0.01. We expect to find pathways associated with tumor aggressiveness, since the astrocytoma grade II is more aggressive than the oligodendroglioma grade II. In fact, for *α* = 0.01, all sets identified only by the spectral distribution test are associated with tumor aggressiveness in different types of cancer. In particular, the REACTOME ACTIVATED NOTCH1 TRANSMITS SIGNAL TO THE NUCLEUS pathway plays an important role in the development of the central nervous system and influences the differentiation of astrocytes. It is also related to cell proliferation and apoptosis in gliomas [28], can promote glioma cell migration and invasion [29], and has already been described as a potential target to glioma therapy [30]. Besides the glioma, many other tumors are associated with the dysregulation of the Notch signaling, such as hepatocellular carcinoma, and lung, breast, pancreatic, and cervical cancer [30]. That pathway presented a large p-value for the GSEA test (p-value = 0.9765), which suggests that this set does not present significant changes in average expression but only in co-expression.

**Table 4:** Comparison of the method findings in a real dataset. Gene sets identified by only one of the three methods (CoGA, GSCA, and GSNCA) and the corresponding p-values. For each group of gene sets, the column in bold indicates the method that identified those sets. The last column shows the p-value obtained by a differential expression analysis tool (GSEA).

Gene set	CoGA	GSCA	GSNCA	GSEA
REACTOME_GROWTH_HORMONE_RECEPTOR_SIGNALING	**0.0097**	0.0900	0.5978	0.1292
REACTOME_ACTIVATED_NOTCH1_TRANSMITS_SIGNAL_TO_THE_NUCLEUS	**0.0019**	0.0144	0.4015	0.9765
REACTOME_ION_CHANNEL_TRANSPORT	**0.0085**	0.0420	0.4852	0.6802
REACTOME_INNATE_IMMUNE_SYSTEM	**0.0082**	0.0124	0.6088	0.1265
BIOCARTA_ERK_PATHWAY	0.0967	**0.0038**	0.0654	0.8653
BIOCARTA_NO1_PATHWAY	0.0592	**0.0074**	0.0439	0.4720
REACTOME_TRANSPORT_TO_THE_GOLGI_AND_SUBSEQUENT_MODIFICATION	0.0146	**0.0087**	0.1536	0.1815
PID_AMB2_NEUTROPHILS_PATHWAY	0.0154	**0.0068**	0.3257	0.0198
REACTOME_O_LINKED_GLYCOSYLATION_OF_MUCINS	0.0173	**0.0094**	0.1542	0.0384
PID_TAP63PATHWAY	0.0967	**0.0079**	0.0836	0.5938
KEGG_LEISHMANIA_INFECTION	0.0149	**0.0041**	0.0946	0.0484
PID_RETINOIC_ACID_PATHWAY	0.4005	0.0676	**0.0096**	0.4325
REACTOME_GLUCONEOGENESIS	0.2123	0.1030	**0.0056**	0.6104
REACTOME_INWARDLY_RECTIFYING_K_CHANNELS	0.1972	0.0124	**0.0092**	0.3969
KEGG_PRIMARY_IMMUNODEFICIENCY	0.1557	0.0564	**0.0043**	0.0331
KEGG_RNA_DEGRADATION	0.4907	0.2708	**0.0051**	0.0644
REACTOME_SIGNALING_BY_NOTCH1	0.5179	0.1438	**0.0031**	0.7173
REACTOME_INTEGRIN_CELL_SURFACE_INTERACTIONS	0.0492	0.0267	**0.0045**	0.1435
REACTOME_INSULIN_RECEPTOR_SIGNALLING_CASCADE	0.2134	0.2027	**0.0057**	0.6336

Other sets identified only by CoGA for *α* = 0.01 are also associated with tumor aggressiveness. The REACTOME GROWTH HORMONE RECEPTOR SIGNALING, REACTOME ION CHANNEL TRANSPORT, REACTOME INNATE IMMUNE SYSTEM pathways are related to, respectively, cellular proliferation, energetic metabolism, and inflammation. Again, the GSEA p-values for those sets were larger than 0.1, indicating that they do not present significant changes in the average expression.

For *α* = 0.05, other sets related to tumor aggressiveness were detected only by CoGA. Examples include gene sets associated with cell proliferation (REACTOME FGFR LIGAND BINDING AND ACTIVATION, REACTOME SMAD2 SMAD3 SMAD4 HETEROTRIMER REGULATES TRANSCRIPTION, PID WNT SIGNALING PATHWAY, and KEGG OOCYTE MEIOSIS pathways), energetic metabolism (REACTOME AMINE COMPOUND SLC TRANSPORTERS pathway), and inflammation (PID CD8TCRPATHWAY, PID IL1PATHWAY, PID IL2 1PATHWAY, PID IL12 2PATHWAY, REACTOME SIGNALING BY ILS, KEGG NATURAL KILLER CELL MEDIATED CYTOTOXICITY, and REACTOME CYTOKINE SIGNALING IN IMMUNE SYSTEM pathways). Those results suggest that CoGA is able to identify gene sets associated with cancer that both GSCA and GSNCA failed to detect.

### CoGA features to analyze a single gene set

In this section we illustrate features available in the CoGA package to explore the properties of a given gene set. The dataset used for this example is the same described in the previous paragraphs and in the Materials and Methods Section, which contains expression data from astrocytoma grade II (AII) and oligondendroglioma grade II (ODII).

The gene set illustrated in this section is the REACTOME ACTIVATED NOTCH1 TRANSMITS SIGNAL TO THE NUCLEUS from the MSigDB (we abbreviate it by RANTSN), which presented the lowest p-value (*p* = 0.0019) by the CoGA (spectral distribution test), and has already been described as associated with glioma aggressiveness. We explain each of the CoGA features (network visualization, gene set properties, gene scores, and gene expression analysis) for the analysis of a single gene set in the paragraphs below.

#### Network visualization

**Fig 3:**
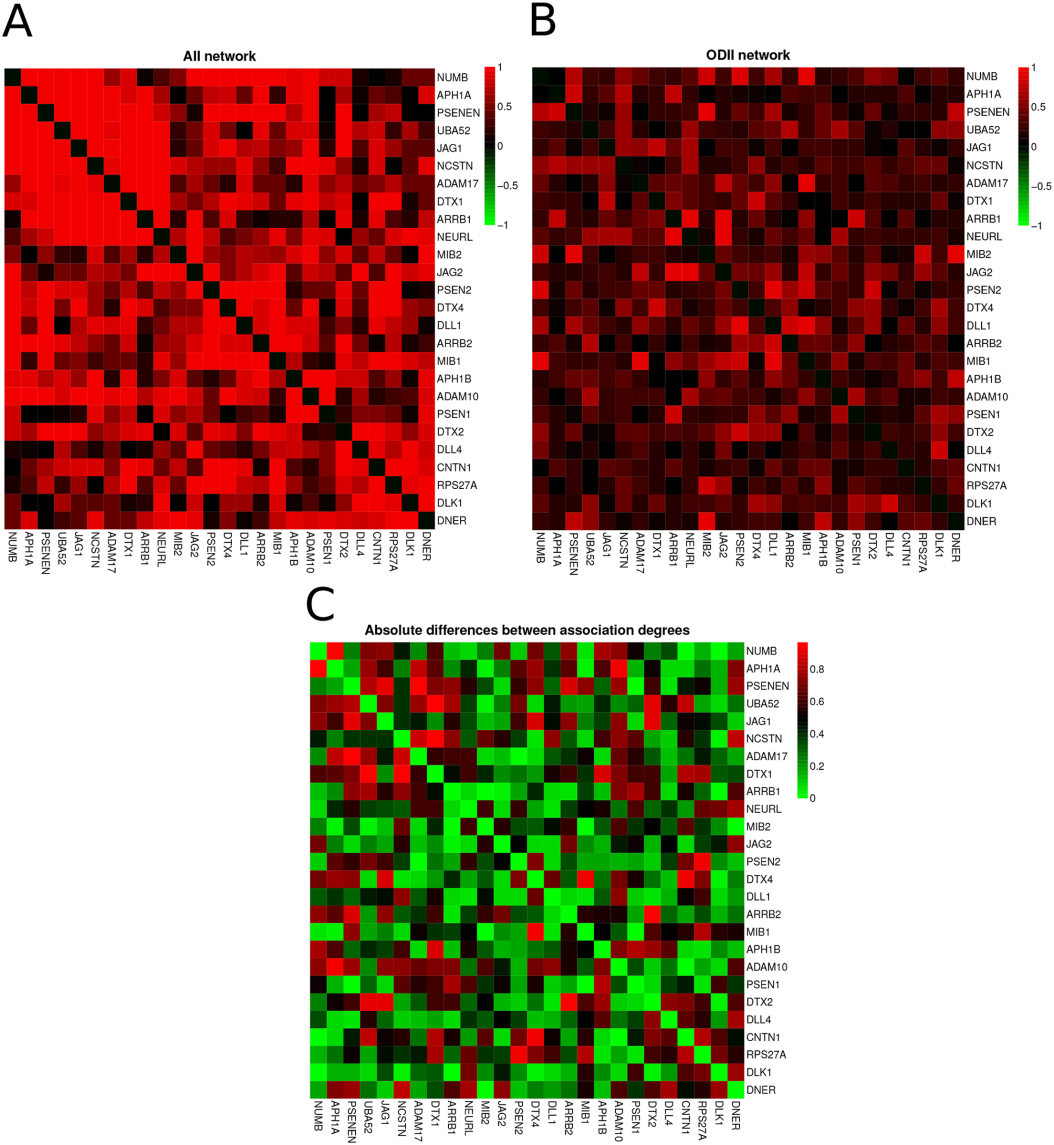
REACTOME ACTIVATED NOTCH1 TRANSMITS SIGNAL TO THE NUCLEUS (RANTSN) gene co-expression graphs visualization: (A) astrocytoma grade II network, which we abbreviate by AII network; (B) oligodendroglioma grade II network, which we abbreviate by ODII network; and (C) differences between AII and ODII networks. In (A) and (B) the red color indicates a high association degree between the row and column genes, while the black color indicates a low association. Matrices (A) and (B) correspond to astrocytoma grade II and oligodendroglioma grade II, respectively. In (C) red, green, and black colors represent, respectively, high, low and intermediate differences between the AII and ODII association degrees.

The network visualization tool shows a matrix of the association degrees between the gene expression levels for each biological condition (AII and ODII, in this example) and a matrix of the differences between them, as illustrated in Fig 3. Those matrices suggest that there are high differences between the edge weights in AII and ODII.

#### Gene set properties

The gene set properties available for weighted networks are average degree centrality, average eigenvector centrality, average clustering coefficient, and spectral entropy. In this example, the RANTSN network has an average degree of 16.90 in astrocytoma grade II and an average degree of 8.19 in oligodendroglioma grade II. The high differences between the gene degrees are in accordance with our differential network analysis.

#### Gene scores

CoGA gene score tool ranks the genes according to their importance in the network. The available measures of “importance” for weighted networks are the degree and eigenvector centralities, and the clustering coefficient (for unweighted networks the betweenness and the closeness centralities are also available).

In this example, the gene with highest degree centrality in the RANTSN astrocytoma grade II network is DTX1 (degree = 19.76), which is a regulator of the Notch signaling pathway. That gene also presented the highest difference in the degree centrality between AII and ODII (difference of 12.70). However it did not show significant difference in the average expression (t-test p-value = 0.06) nor in the median expression (Wilcoxon-Mann-Whitney p-value = 0.057) at a p-value threshold of 0.05. Interestingly, the expression of the gene DTX1 is correlated with patients survival in gliomas, and its over-expression can increase cell migration and invasion in glioblastoma multiforme [31]. This regulator gene can also induce pathways to protect tumor cells from apoptosis and to stimulate the cell proliferation [31]. Therefore, DTX1 is highly associated with tumor cell aggressiveness.

In the RANTSN oligodendroglioma grade II network, the gene with highest degree centrality is DLL1 (degree = 10.86), which acts as a ligand for Notch receptors.

#### Gene expression analysis

**Fig 4:**
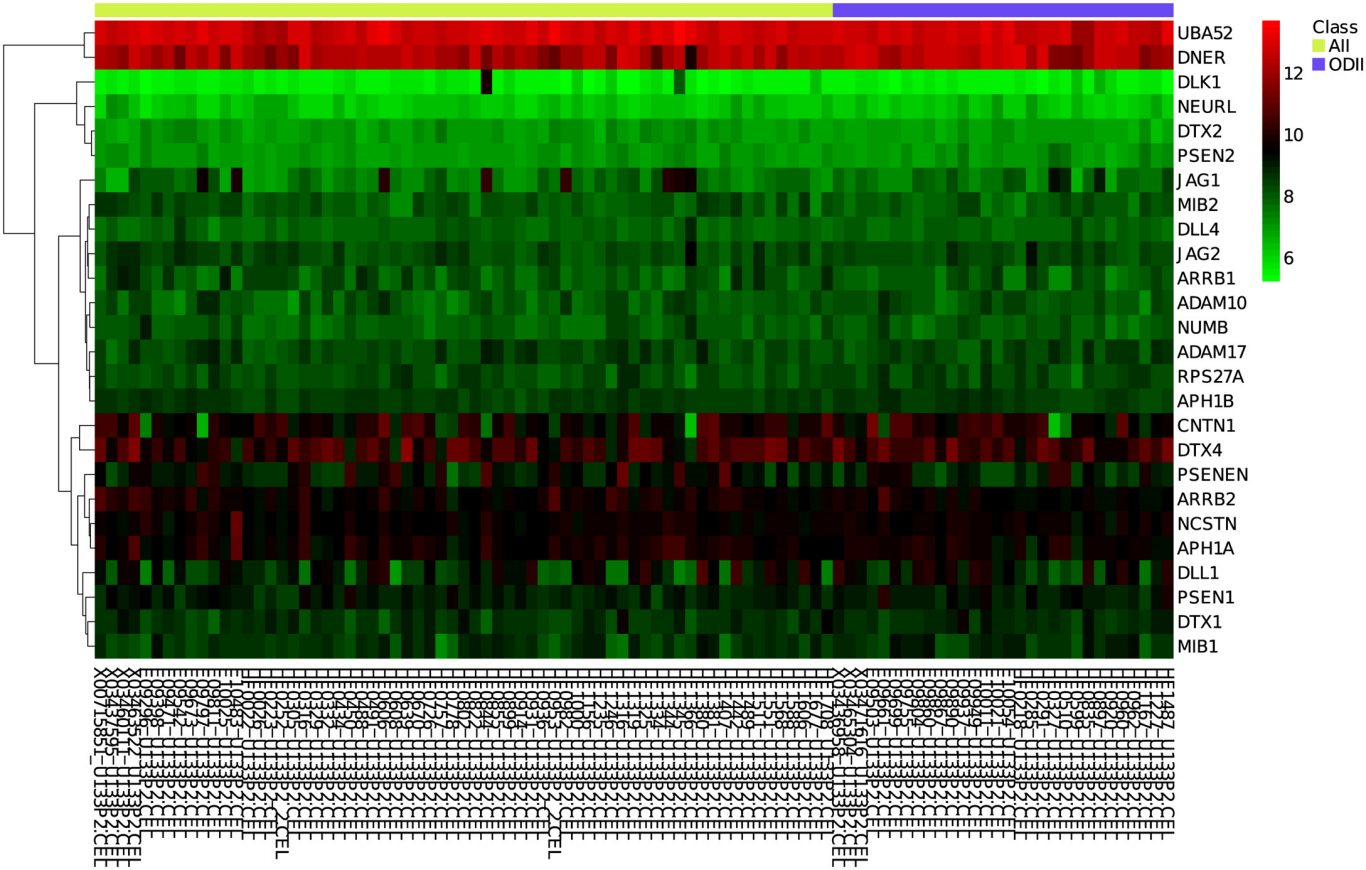
REACTOME ACTIVATED NOTCH1 TRANSMITS SIGNAL TO THE NUCLEUS (RANTSN) gene expression heatmap. Heatmap showing the expression levels of genes belonging to the REACTOME ACTIVATED NOTCH1 TRANSMITS SIGNAL TO THE NUCLEUS pathway in astrocytoma grade II (green label) and oligodendroglioma grade II (blue label) microarrays. The red, black, and green colors on the expression matrix represent, respectively, the highest, intermediate, and lowest expression levels.

CoGA also includes the standard single gene differential expression analysis. The tool shows the gene expression heatmap (Fig 4), the result of the t-test (difference in means) and the Wilcoxon-Mann-Whitney test (difference in medians). The expression heatmap of the RANTSN set did not reveal visual differences between AII and ODII. Only the ARRB2 gene had t-test nominal p-value less than 5%, and only ARRB2 and DNER had Wilcoxon-Mann-Whitney nominal p-values less than 5%.

#### Final considerations about the further analysis

As discussed previously, the RANTSN was detected by the spectral distribution test, but was not detect by the GSEA tool, which performs differential expression analysis. In accordance with those results, this example of CoGA single gene set analysis revealed “important” genes from the RANTSN set that are differentially co-expressed but are not differentially expressed between AII and ODII. Therefore CoGA single gene set analysis might be helpful in the identification of key genes in a disease, by complementing the standard differential expression analysis.

## Conclusion

We present CoGA, an R package, which (i) performs differential co-expression network analyses; (ii) compares underexplored network features and also several standard structural properties; and (iii) carries out statistical tests to estimate the significance of the results. We have shown that all the statistical tests performed by CoGA effectively control the rate of false positives. Our simulation experiments and applications in real dataset suggest that CoGA complements previous tools for differential co-expression analysis (GSCA and GSNCA). Numerical results combined with visual inspection in the graphical user interface might be helpful in the identification of key sets of genes.

## Availability and Requirements

- CoGA home page: www.ime.usp.br/˗suzana/coga.
- Download page: https://sourceforge.net/projects/coga/.
- Operating system(s): Platform Independent.
- Other requirements: R ≥ 3.0.0, R packages (shiny ≥ 0.8.0, WGCNA, igraph, shinyBS, RColorBrewer, Hmisc, psych, RJSONIO, whisker, yaml, pheatmap, ggplot2)

## Supporting Information

S1 Table MSigDB canonical pathways differential network analysis. (XLS)

## References

[1] Subramanian A, Tamayo P, Mootha VK, Mukherjee S, Ebert BL, Gillette MA, et al Gene set enrichment analysis: A knowledge-based approach for interpreting genome-wide expression profiles. PNAS. 2005 10;102(43):15545–15550. 10.1073/pnas.0506580102 16199517 PMC1239896

[2] Kato K, Toki T, Shimizu M, Shiozawa T, Fujii S, Nikaido T, et al Expression of replication-licensing factors MCM2 and MCM3 in normal, hyperplastic, and carcinomatous endometrium: correlation with expression of Ki-67 and estrogen and progesterone receptors. Int J Gynecol Pathol. 2003;22(4):334–340. 14501812 10.1097/01.pgp.0000092129.10100.5e

[3] Chan WY, Cheung KK, Schorge JO, Huang LW, Welch WR, Bell DA, et al Bcl-2 and p53 protein expression, apoptosis, and p53 mutation in human epithelial ovarian cancers. Am J Pathol. 2000;156(2):409–417. 10.1016/S0002-9440(10)64744-X 10666369 PMC1850061

[4] Keller MP, Choi Y, Wang P, Davis DB, Rabaglia ME, Oler AT, et al A gene expression network model of type 2 diabetes links cell cycle regulation in islets with diabetes susceptibility. Genome Res. 2008;18(5):706–716. 10.1101/gr.074914.107 18347327 PMC2336811

[5] Hudson NJ, Reverter A, Dalrymple BP. A Differential Wiring Analysis of Expression Data Correctly Identifies the Gene Containing the Causal Mutation. PLoS Comput Biol. 2009;5(5):e1000382 10.1371/journal.pcbi.1000382 19412532 PMC2671163

[6] de la Fuente A. From ‘differential expression’ to ‘differential networking’—identification of dysfunctional regulatory networks in diseases. Trends in Genetics. 2010;26(7):326–333. 10.1016/j.tig.2010.05.001 20570387

[7] Yang J, Yu H, Liu BH, Zhao Z, Liu L, Ma LX, et al DCGL v2.0: An R Package for Unveiling Differential Regulation from Differential Co-expression. PLoS ONE. 2013 11;8(11):e79729 10.1371/journal.pone.0079729 24278165 PMC3835854

[8] Liu BH, Yu H, Tu K, Li C, Li YX, Li YY. DCGL: an R package for identifying differentially coexpressed genes and links from gene expression microarray data. Bioinformatics. 2010 10;26(20):2637–2638. 10.1093/bioinformatics/btq471 20801914 PMC2951087

[9] Yu H, Liu BH, Ye ZQ, Li C, Li YX, Li YY. Link-based quantitative methods to identify differentially coexpressed genes and gene Pairs. BMC Bioinformatics. 2011 8;12(1):315 10.1186/1471-2105-12-315 21806838 PMC3199761

[10] Watson M. CoXpress: differential co-expression in gene expression data. BMC Bioinformatics. 2006 11;7(1):509 10.1186/1471-2105-7-509 17116249 PMC1660556

[11] Tesson BM, Breitling R, Jansen RC. DiffCoEx: a simple and sensitive method to find differentially coexpressed gene modules. BMC Bioinformatics. 2010 10;11(1):497 10.1186/1471-2105-11-497 20925918 PMC2976757

[12] Amar D, Safer H, Shamir R. Dissection of Regulatory Networks that Are Altered in Disease via Differential Co-expression. PLoS Comput Biol. 2013 3;9(3):e1002955 10.1371/journal.pcbi.1002955 23505361 PMC3591264

[13] Langfelder P, Horvath S. WGCNA: an R package for weighted correlation network analysis. BMC Bioinform. 2008 12;9(1):559 10.1186/1471-2105-9-559 PMC2631488 19114008

[14] Choi Y, Kendziorski C. Statistical methods for gene set co-expression analysis. Bioinformatics. 2009 1;25(21):2780–2786. 10.1093/bioinformatics/btp502 19689953 PMC2781749

[15] Rahmatallah Y, Emmert-Streib F, Glazko G. Gene Sets Net Correlations Analysis (GSNCA): a multivariate differential coexpression test for gene sets. Bioinformatics. 2014 2;30(3):360–368. 10.1093/bioinformatics/btt687 24292935 PMC4023302

[16] Barabási AL, Oltvai ZN. Network biology: understanding the cell’s functional organization. Nat Rev Genet. 2004 2;5(2):101–113. 10.1038/nrg1272 14735121

[17] Shannon P, Markiel A, Ozier O, Baliga NS, Wang JT, Ramage D, et al Cytoscape: A Software Environment for Integrated Models of Biomolecular Interaction Networks. Genome Res. 2003 1;13(11):2498–2504. 10.1101/gr.1239303 14597658 PMC403769

[18] Takahashi DY, Sato JaR, Ferreira CE, Fujita A. Discriminating Different Classes of Biological Networks by Analyzing the Graphs Spectra Distribution. PLoS ONE. 2012 12;7(12):e49949 10.1371/journal.pone.0049949 23284629 PMC3526608

[19] Pearson K. Notes on the History of Correlation. Biometrika. 1920 10;13(1):25–45. 10.1093/biomet/13.1.25

[20] Spearman C. The Proof and Measurement of Association between Two Things. Am J Psychol. 1904 1;15(1):72–101. 10.2307/1412159 3322052

[21] Kendall MG. A New Measure of Rank Correlation. Biometrika. 1938 1;30(1–2):81–93. 10.2307/2332226

[22] Benjamini Y, Hochberg Y. Controlling the False Discovery Rate: A Practical and Powerful Approach to Multiple Testing. J R Stat Soc Ser B Stat Methodol. 1995;57(1):289–300.

[23] Van Mieghem P. Graph Spectra for Complex Networks. Cambridge: Cambridge University Press; 2010.

[24] Sturges HA. The Choice of a Class Interval. J Am Statist Assoc. 1926;21(153):65–66. 10.1080/01621459.1926.10502161

[25] Silverman BW. Density Estimation for Statistics and Data Analysis. Boca Raton: Chapman and Hall; 1986.

[26] Irizarry RA, Hobbs B, Collin F, Beazer-Barclay YD, Antonellis KJ, Scherf U, et al Exploration, normalization, and summaries of high density oligonucleotide array probe level data. Biostatistics. 2003 4;4(2):249–264. 10.1093/biostatistics/4.2.249 12925520

[27] Dai M, Wang P, Boyd AD, Kostov G, Athey B, Jones EG, et al Evolving gene/transcript definitions significantly alter the interpretation of GeneChip data. Nucleic Acids Res. 2005;33(20):e175 10.1093/nar/gni179 16284200 PMC1283542

[28] Purow BW, Haque RM, Noel MW, Su Q, Burdick MJ, Lee J, et al Expression of Notch-1 and its ligands, Delta-like-1 and Jagged-1, is critical for glioma cell survival and proliferation. Cancer Res. 2005 3;65(6):2353–2363. 10.1158/0008-5472.CAN-04-1890 15781650

[29] Zhang X, Chen T, Zhang J, Mao Q, Li S, Xiong W, et al Notch1 promotes glioma cell migration and invasion by stimulating *β*-catenin and NF-*κ*B signaling via AKT activation. Cancer Sci. 2012;103(2):181–190. 10.1111/j.1349-7006.2011.02154.x 22093097

[30] Stockhausen MT, Kristoffersen K, Poulsen HS. The functional role of Notch signaling in human gliomas. Neuro Oncol. 2010 1;12(2):199–211. 10.1093/neuonc/nop022 20150387 PMC2940575

[31] Huber RM, Rajski M, Sivasankaran B, Moncayo G, Hemmings BA, Merlo A. Deltex-1 Activates Mitotic Signaling and Proliferation and Increases the Clonogenic and Invasive Potential of U373 and LN18 Glioblastoma Cells and Correlates with Patient Survival. PLoS ONE. 2013 2;8(2):e57793 10.1371/journal.pone.0057793 23451269 PMC3581491

